# The Association of Maternal Age With Fetal Growth and Newborn Measures: The Mumbai Maternal Nutrition Project (MMNP)

**DOI:** 10.1177/1933719118799202

**Published:** 2018-11-12

**Authors:** Chiara Di Gravio, Ashwin Lawande, Ramesh D. Potdar, Sirazul A. Sahariah, Meera Gandhi, Nick Brown, Harsha Chopra, Harshad Sane, Sarah H. Kehoe, Ella Marley-Zagar, Barrie M. Margetts, Alan A. Jackson, Caroline H. D. Fall

**Affiliations:** 1 MRC Lifecourse Epidemiology Unit, University of Southampton, Southampton General Hospital, Southampton, United Kingdom; 2 Dr Joshi Imaging Clinic, Mumbai, India; 3 Centre for the Study of Social Change, Mumbai, India; 4 International Centre for Maternal and Child Health, Akademia Sjukhuset, University of Uppsala MTC-huset, Sweden; 5 Public Health Nutrition, University of Southampton, Southampton, United Kingdom; 6 NIHR Southampton Biomedical Research Centre, Southampton, United Kingdom

**Keywords:** fetal biometry, ultrasound, maternal age, pregnancy, newborn, India

## Abstract

**Background:**

Young maternal age is associated with poorer birth outcomes, but the mechanisms are incompletely understood. Using data from a prospective cohort of pregnant women living in Mumbai slums, India, we tested whether lower maternal age was associated with adverse fetal growth.

**Methods:**

Fetal crown-rump length (CRL) was recorded at a median (interquartile range, IQR) of 10 weeks’ gestation (9-10 weeks). Head circumference (HC), biparietal diameter (BPD), femur length (FL), and abdominal circumference (AC) were recorded at 19 (19-20) and 29 (28-30) weeks. Newborns were measured at a median (IQR) of 2 days (1-3 days) from delivery. Gestation was assessed using prospectively collected menstrual period dates.

**Results:**

The sample comprised 1653 singleton fetuses without major congenital abnormalities, of whom 1360 had newborn measurements. Fetuses of younger mothers had smaller CRL (0.01 standard deviation [SD] per year of maternal age; 95% confidence interval CI: 0.00-0.02^1^; *P* = .04), and smaller HC, FL, and AC at subsequent visits. Fetal growth of HC (0.04 cm; 95% CI: 0.02-0.05; *P* < .001), BPD (0.01 cm; 95% CI: 0.00-0.01; *P* = .009), FL (0.04 cm; 95% CI: 0.02-0.06; *P* < .001), and AC (0.01 cm; 95% CI: 0.00-0.01; *P* = .003) up to the third trimester increased with maternal age. Skinfolds, head, and mid-upper arm circumferences were smaller in newborns of younger mothers. Adjusting for maternal prepregnancy socioeconomic status, body mass index, height, and parity attenuated the associations between maternal age and newborn size but did not change those with fetal biometry.

**Conclusion:**

Fetuses of younger mothers were smaller from the first trimester onward and grew slower, independently of known confounding factors.

## Introduction

Young (≤19 years) and advanced (≥35 years) maternal age during pregnancy has been linked to adverse fetal and birth outcomes. Young maternal age is associated with an increased risk of fetal growth restriction, preterm delivery, low birth weight (LBW), small for gestational age (SGA), and neonatal mortality.^2–5^ Advanced maternal age is associated with higher perinatal mortality and an increased risk of intrauterine growth restriction, LBW, and preterm delivery.^6–8^ These associations are consistent and, thought incompletely understood, are thought to arise from biological and social factors. Many younger mothers are still growing, and their nutritional needs compete with those of the fetus.^3,9^ Younger mothers are less likely to seek prenatal care and more likely to be primiparous and to be of lower socioeconomic status.^3^ Older mothers are at higher risk of gestational diabetes and preeclampsia,^6,10^ which can impair fetal development. In high-income countries, older mothers tend to be better educated of higher socioeconomic status and lower parity; whereas in low and middle-income countries, older mothers are likely to have higher parity and live in a more deprived environment.^2^

Numerous studies have looked at the relationship between maternal age and pregnancy outcomes in both high- and low-middle income countries. However, literature on associations of maternal age with fetal size and growth is scarce. Newborns can attain the same size/weight via different fetal growth trajectories, and it is important to understand how and when in gestation, maternal age may affect fetal growth.

We have used data from a group of women living in Mumbai, India, to (1) assess associations of maternal age at conception with fetal size/growth and newborn measures and (2) examine whether maternal prepregnancy body mass index (BMI), height, parity, diet, tobacco use, weight gain in pregnancy, and socioeconomic status partially explained any associations. These women were taking part in a randomized controlled trial of a preconceptional nutritional intervention (a daily micronutrient-rich snack), which increased newborn birth weight when the mother was supplemented for >3 months before conception but had no effect on fetal size or growth.^12^

## Materials and Methods

The data were collected as part of the Mumbai Maternal Nutrition Project, a randomized controlled trial investigating the effect on newborn measures of a food-based micronutrient-rich supplement taken from before pregnancy until delivery. Enrollment in the trial took place between 2006 and 2012. Women living in slums covered by the health and social programs of the nongovernmental organization the Centre for the Study of Social Change (CSSC) were eligible if they were aged <40 years, married, nonpregnant, not sterilized, and planned to have children and to deliver in Mumbai. Women were randomized to receive either a daily micronutrient-rich snack containing green leafy vegetables, fruit, and milk or a snack containing foods of low-micronutrient content such as potato and onion, in addition to their normal diet. Further information on the trial can be found elsewhere.^11^ We have previously shown that the intervention increased birth weight and other “soft tissue” measurement (skinfolds and abdominal, mid-upper arm, and chest circumference) in the newborns of mothers supplemented for >3 months before pregnancy,^11^ but there were no differences in fetal measurements between intervention and control groups.^12^

### Data Collection

Health workers made home visits to explain the trial, and community meetings were held to answer questions and obtain consent. Women were screened for eligibility and individual written informed consent was obtained. At recruitment, weight and height were measured and information on women’s occupation, education, parity, and tobacco use (both in chewed and smoked form) were recorded. Women’s socioeconomic status was assessed using the Standard of Living Index (SLI), which is based on housing type, utilities, and household possessions.^13^ A higher SLI score indicates a higher socioeconomic status. Diet was assessed at recruitment and in the second trimester of pregnancy using a food frequency questionnaire.^14^

The snacks were prepared fresh daily, were provided 6 days per week, and staff at the supplementation centers observed and recorded their intake. Center staff also recorded the women’s serial menstrual period dates. Women who missed 2 periods had a urinary pregnancy test and, if it was positive, were invited to a central clinic at CSSC at 9 to 12 weeks of gestation for an obstetric assessment. Supplementation continued throughout pregnancy.

Fetal biometry was determined by ultrasound (Siemens Sonoline ADARA with a 4 MHz probe) at 3 time points during pregnancy corresponding to 9 to 12, 19 to 21, and 28 to 32 weeks of gestation, respectively. Measurements were performed by a single operator (AL) using standard techniques.^15^ At visit 1, crown-rump length (CRL) was measured. If women attended late (>13 weeks of gestation), fetal head circumference (HC), biparietal diameter (BPD), femur length (FL), and abdominal circumference (AC) were recorded instead. Head circumference, BPD, FL, and AC were assessed at the 2 subsequent visits. Head circumference was calculated using the longest and shortest axes of the fetal head, measured from the outer to outer surfaces of the skull. Biparietal diameter was measured from outer to inner surfaces of the skull. Femur length was measured along the long axis of the femur without the distal femoral epiphysis. Abdominal circumference was estimated using the anteroposterior and the transverse diameters,^15^ after ensuring that the stomach bubble was visible, the abdomen filled at least 30% of the monitor screen, and neither the kidneys or the bladder were visible.^16^ At each examination HC, BPD and FL were measured once, whereas AC was taken in triplicate and the best or the average of the 3 measures, assessed by the operator, was used in the analysis.^12^ At visits 2 and 3, using one of the Hadlock formula,^17^ we computed the estimated fetal weight (EFW) as log_10_ (EFW) = 1.326 − 0.00326 × AC × FL + 0.0107 × HC + 0.0438 × AC + 0.158 × FL.

For the purpose of this study, in which we wanted to detect possible relationships between maternal age and fetal size, even in the early stages of pregnancy, we based gestational age on the last menstrual period (LMP) date rather than deriving gestational age from a size measurement using the ultrasound data. Throughout the trial, health workers maintained a record of the women’s LMP dates and updated this every month.

Newborns were measured within 10 days after birth using standardized techniques. Trained research nurses measured weight and length, head, mid-upper arm, chest and abdomen circumference, and triceps and subscapular skinfolds.^10^ For each newborn, weight and length were measured once, whereas circumferences and skinfolds were taken in triplicate and averaged. Gestation was assessed using prospectively collected menstrual period date. Preterm birth was defined as gestational age <37 weeks, and SGA as birth weight below the age-and-sex-specific 10th percentile of the INTERGROWTH 21st standards.^18^ Complete information on data collection can be found elsewhere.^10^

### Exposures and Outcomes

The primary exposure was maternal age at conception. This was calculated as the difference between the woman’s date of birth and LMP date. Primary outcomes for the current analysis were fetal size/growth at different stages of pregnancy and newborn measures. Secondary outcomes were gestational age and risk of preterm and SGA birth. We included tobacco use, weekly intakes of milk and green leafy vegetables in pregnancy, SLI score, maternal prepregnancy BMI, height, and parity as covariates in the model; however, some of those variables could be potential mediators. For women with recorded weight during pregnancy, we further looked at whether gestational weight gain explained the associations between maternal age and fetal and newborn measures, by considering weight gain in early pregnancy (difference between weight at the first visit and at registration) and weight gain between first and third visits.

### Analysis Sample

A total of 6513 women were recruited. Initially, pregnancies were followed up only if the women started supplementation at least 3 months prior to their LMP date. However, the exclusion of women who conceived within 3 months of starting supplementation caused disappointment in the community, and from December 2008, we included all pregnancies. In total, 2291 women became pregnant. Pregnancies resulting in abortions, terminations, stillbirths and maternal deaths (n = 269), and those with no information on delivery outcome (n = 22) were excluded. We excluded twins (n = 26), fetuses with major congenital abnormalities (n = 12), and those of unknown sex (n = 41). It is illegal in India to determine the sex of the fetus on ultrasound, and these were cases where the mother had 1 or more ultrasound scans but was then lost to follow-up, and newborn sex was not ascertained.

**Figure 1.**
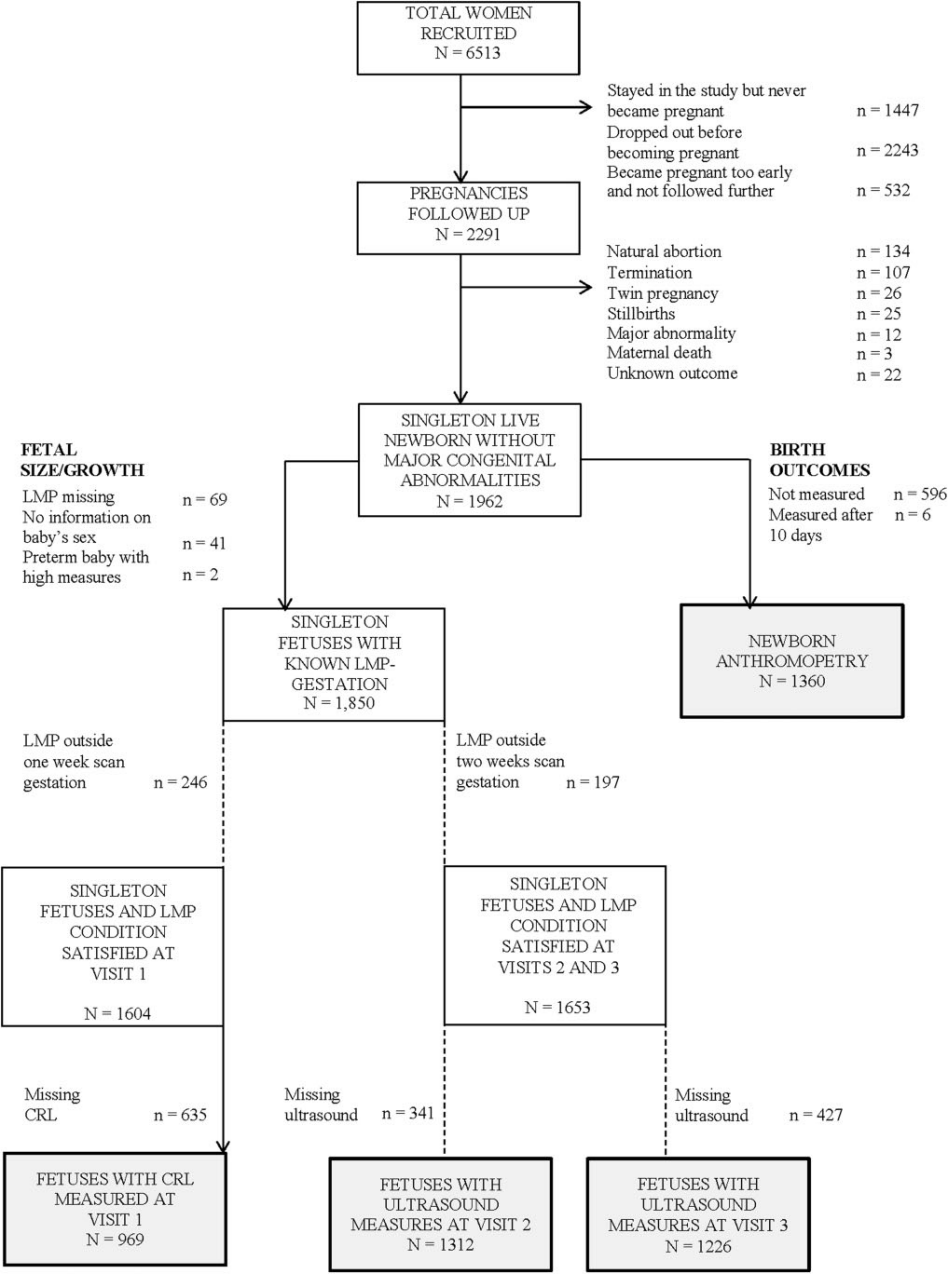
Flowchart of the participants. CRL, crown rump length.

To examine the associations with fetal size/growth, we excluded pregnancies with missing maternal LMP (n = 69). At visit 1, women for whom the LMP-derived gestation differed by more than 1 week from the gestation estimated from an early (<20 weeks) ultrasound scan (n = 246) were excluded as the former was likely to be inaccurate. At visits 2 and 3, we excluded women whose difference between first trimester LMP-derived gestational age and ultrasound-derived gestational age was greater than 2 weeks (n = 197). Two preterm babies whose gestational-age-adjusted fetal measures, at each scan, were >3 standard deviations (SDs) higher than the population mean were excluded because, given their available fetal biometric parameters, their LMP date was likely to be wrong (Figure 1).

Newborn measures were excluded from the analysis only if the baby was measured more than 10 days after birth (n = 6). Exclusions based on LMP date were not employed. This led to a sample of 1360 newborns (Figure 1).

### Statistical Methods

**Figure 2.**
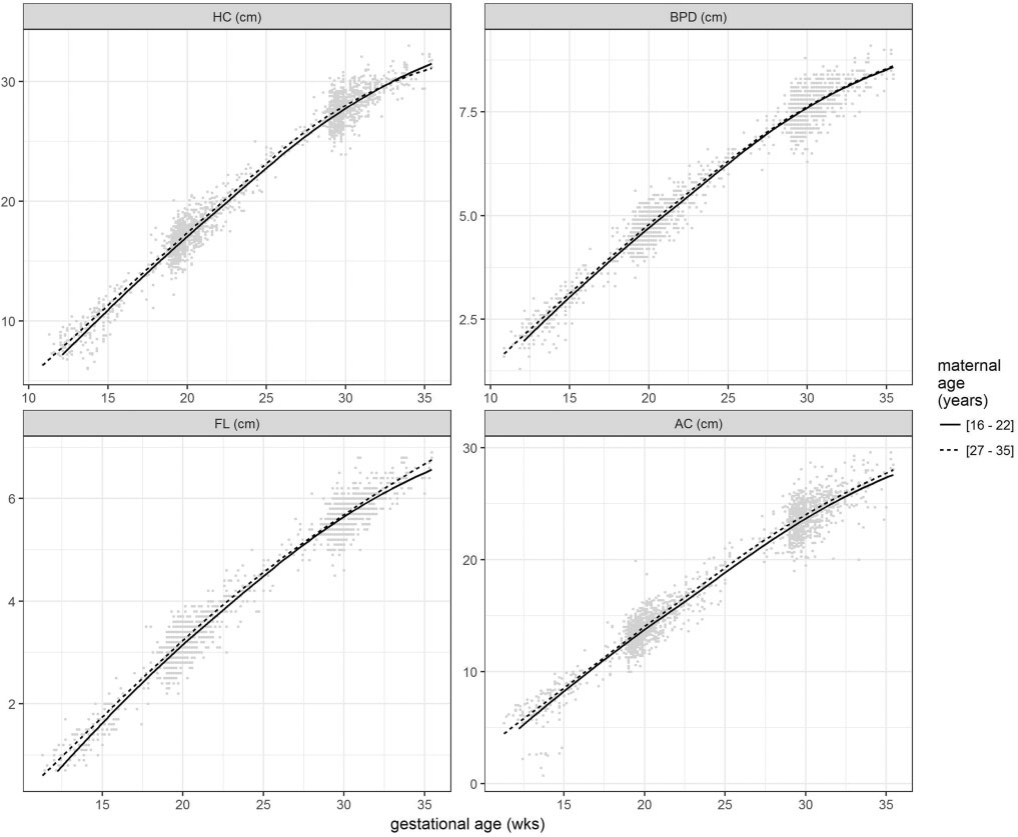
Plots of HC, BPD, AC, and FL according to gestational age (weeks) and lowest and highest tertiles of maternal age at conception. The continuous line represents the mean growth trend of fetuses whose mothers were in the lowest tertile of maternal age (age 22 or less), whereas the dashed line summarizes the mean growth trend of fetuses of mothers who are in the upper tertile of maternal age (age 27 and more). AC, abdominal circumference; BPD, biparietal diameter; FL, femur length; HC, head circumference.

Maternal age was used as a continuous variable in all models and as a categorical variable for Figure 2. To account for differences in fetal size between sexes, and for varying gestational ages at each visit, we transformed fetal ultrasound measures into internal sex-and-gestational-age-adjusted *z* scores using the LMS method.^19^ Crown-rump length was analyzed in the complete sample and also in a subgroup of women with regular menstrual cycle length (defined as within 28 ± 4 days^5^). To account for different timing of ovulation, median cycle length was added as a possible covariate in the subgroup analysis.^5^ Birth measures were converted into *z* scores after adjusting for sex and gestational age at delivery. We examined differences in baseline measurements between age groups using χ^2^ tests, analysis of variance, and Kruskal–Wallis tests for categorical, normally and nonnormally distributed continuous variables, respectively. We inspected possible differences in weekly intakes of green leafy vegetables, milk, and fruit before and during pregnancy using Wilcoxon signed-rank test. We compared the fetal size at each visit with the median INTERGROWTH 21st standards^16^ using multiple Mann–Whitney tests.

To analyze associations between maternal age and continuous and binary measures of fetal size and birth outcomes, we used a series of linear or logistic regression models as appropriate. First, we adjusted for allocation group only (model 1); then, we adjusted for potential confounders, including tobacco use and weekly intakes of green leafy vegetables and milk in pregnancy, SLI score, parity, maternal prepregnancy BMI, and height (model 2). Women’s fruit intakes in pregnancy, occupation, and education were initially considered as possible confounders; however, as they did not modify the observed associations, or improve the models’ goodness of fit, we did not include them in the analyses presented.

Considering the subset of women whose weight in pregnancy was recorded, we used model 3, further adjusted for weight gain during pregnancy, to study the effect of gestational weight gain on the associations between maternal age and fetal and newborn size.

Associations between maternal age and fetal growth up to the third trimester were analyzed using mixed-effects models to account for the correlation between repeated observations in the same fetuses and for the possibility of a nonlinear association between fetal growth and gestational age. First, we implemented 4 models (1 for each fetal biometry measured longitudinally), where the raw fetal sizes recorded at different trimesters were included as outcome, and maternal age, gestational age, and sex as predictors. Afterward, we carried out a series of models with adjustment similar to those used for analyzing the associations of maternal age with fetal and newborn size. To relax the assumptions on the trend between fetal outcomes and gestational age, and to account for the small number of women in the younger age-group, we tested the same associations using restricted cubic splines with knots fixed at percentiles of unique ages. However, as the results of the cubic splines were similar to those of the linear mixed model, only the latter is presented. Results were considered statistically significant when *P* < .05. The analyses were performed using R V.3.4.1^20^ and Stata V.14 (Stata Corporation, College Station, Texas).

### Ethics

The trial (ISRCTN62811278) was granted ethics permission by the committees of BYL Nair and TN Medical College, Grant Medical College, and Sir JJ Group of Hospitals, Mumbai, and by the ethics committees of the Hampshire and Isle of Wight Strategic Health authority. An independent data-monitoring committee reviewed the data every 6 months for 2 years and then annually. The trial protocol can be obtained from the corresponding author.

## Results

The median age at conception was 25 years (interquartile range, IQR: 22-28 years, range: 16-37 years). Younger women were lighter, had lower BMI, socioeconomic status, educational attainment and were less likely to be in paid work (Table 1). At recruitment, they had lower weekly intakes of milk and fruit. The percentage of underweight (BMI ≤ 18.5 kg/m^2^) women decreased with age: dropping from 42% in the youngest mothers to 24% in the oldest group. The percentage of overweight and obese (BMI > 25 kg/m^2^) women rose from 5% in the youngest mothers to 22% in those aged 30 and more. Percentages of Muslims and Hindi speakers were highest among women who were ≤19 and decreased with age. Younger mothers gained, on average, less weight in early pregnancy and more weight between the first and third trimesters (Table 1).

**Table 1.** Baseline (Prepregnancy) Characteristics of Women Who Became Pregnant and Their Diet Weight Gain During Pregnancy According to Tertiles of Age at Conception.^a^

	All		≤22 Years		23 to 26 Years		27 Years and Over		P^b^
	Median (IQR) or n(%)	N	Median (IQR) or n(%)	N	Median (IQR) or n(%)	N	Median (IQR) or n(%)	N	
Data collected prepregnancy									
Weight, kg	45.7 (40.3, 51.8)	2284	44.0 (39.5, 49.6)	861	46.3 (40.6, 52.3)	782	47.8 (42.0, 55.3)	641	<.001
Height, cm^c^	151.4 (5.47)	2284	151.5 (5.73)	862	151.75 (5.22)	781	151.0 (5.40)	641	.10
BMI, kg/m^2^	19.8 (17.9, 22.5)	2283	19.1 (17.5, 21.5)	861	19.9 (18.0, 22.8)	781	20.9 (18.4, 24.1)	641	<.001
Waist, cm^c^	69.9 (9.42)	2283	67.6 (8.00)	862	70.0 (9.24)	781	72.9 (10.5)	640	<.001
Subscapular, mm	21.3 (15.3, 28.6)	2285	19.3 (144, 25.3)	862	21.5 (15.6, 28.8)	782	24.3 (16.2, 34.3)	641	<.001
Triceps, mm	13.5 (10.0, 18.7)	2285	12.4 (9.47, 16.2)	862	14.2 (10.0, 19.2)	782	15.6 (11.2, 21.4)	641	<.001
Previous deliveries		2285		862		782		641	<.001
0	730 (32.0%)		416 (48.6%)		222 (28.4%)		92 (14.4%)		
1	1059 (46.4%)		364 (42.2%)		370 (47.3 %)		325 (50.7%)		
2+	496 (21.7%)		82 (9.51%)		190 (24.3%)		224 (35.0%)		
Prepregnancy weekly frequency of dietary intakes	2285		862		782		641		
Milk and milk products (excluding tea)	1 (0, 2)		0 (0, 2)		1 (0, 2)		1 (0, 2)		.01
Fruit	2 (1, 5)		2 (1, 5)		3 (1, 5)		3 (1, 5)		.02
GLV	1 (1, 3)		1 (0, 3)		1 (1, 3)		2 (1, 3)		.09
Tobacco use	205 (9.0%)		64 (7.4%)		70 (9.0%)		70 (10.9%)		.06
SLI score^c^	24.9 (6.1)	2209	24.1 (6.0)	840	24.9 (6.1)	757	25.9 (5.9)	612	<.001
Religion		2284		861		782		641	<.001
Hindu	1608 (70.4%)		551 (64.0%)		565 (72.3%)		492 (77.7 %)		
Muslim	596 (26.1%)		283 (32.9%)		190 (24.3%)		123 (19.9%)		
Other	80 (3.50%)		27 (3.14%)		27 (3.45%)		46 (4.06%)		
Education		2283		861		782		640	<.001
Primary	244 (10.7%)		102 (11.9%)		82 (10.5 %)		60 (9.38%)		
Secondary	1917 (84.0%)		738 (85.7%)		656 (83.9%)		523 (81.7%)		
Graduate	122 (5.3%)		21 (2.44%)		44 (5.63%)		57 (8.91%)		
Mother tongue		2281		860		782		639	<.001
Marathi	1214 (53.2%)		369 (42.9%)		429 (54.9%)		416 (65.1%)		
Hindi	843 (37.0%)		396 (46.05%)		281 (35.9%)		166 (26.0%)		
Other	224 (9.82%)		95 (11.1%)		72 (9.21%)		57 (8.92%)		
Occupation		2285		862		782		641	<.001
In paid work	480 (21%)		103 (12.0%)		183 (23.4%)		194 (30.3%)		
Not in paid work	1805 (79%)		759 (88.5%)		599 (76.6%)		447 (69.7%)		
Data collected during pregnancy									
Pregnancy weekly frequency of dietary intakes^d^		1566		862		782		641	
Milk and milk products (excluding tea)	2 (0, 7)		1 (0, 4)		1 (0, 7)		1 (0, 6)		<.001
Fruit	5 (2, 9)		2 (0, 6)		3 (0, 7)		2 (0, 6)		.88
GLV	2 (1, 2)		1 (0, 2)		1 (0, 2)		1 (0, 2)		.01
Weight gain between registration and visit 1, kg^c,f^	1.53 (5.71)	1058	1.03 (3.38)	380	1.40 (3.97)	366	1.66 (3.76)	310	.005^e^
Weight gain between visit 1 and visit 3, kg^c,f^	5.68 (3.00)	1003	6.30 (2.80)	358	5.48 (2.69)	348	5.13 (3.43)	297	<.001^e^

Abbreviations: BMI, body mass index; GLV, green leafy vegetables; IQR, interquartile range.

^a^ Five women did not have information on maternal age at conception.

^b^ *P* Values were from χ^2^ tests, *t* tests, and Mann–Whitney *U* tests for categorical, normally and nonnormally distributed continuous variables, respectively.

^c^ For normally distributed variables mean and standard deviation are reported.

^d^ Milk, GLV, and fruit consumption does not include treatment snacks.

^e^ *P* Values for the differences of weight gain during pregnancy were found using linear regression with maternal age at conception as continuous predictor.

^f^ Visit 1 gestational age range: 5 to 19 weeks and visit 3 gestational age range: 21 to 35 weeks.

### Fetal Size and Growth

The median (IQR) gestational age at each examination was 10 (9-12), 19 (19-20), and 29 (28-30) weeks, respectively. Fetal ultrasound measures at each visit are reported in Table 2. Compared with the median INTERGROWTH 21st standard, fetuses had significantly smaller CRL at visit 1 and head and abdominal circumferences at visit 3 (Table 2).

**Table 2.** Summary of Available Fetal Biometry at Each Visit.

	MMNP Fetuses		INTERGROWTH 21st (50th Percentile)		*P*
	Median	IQR	Median	IQR	
Size at visit 1					
CRL, cm	2.9	(2.4-3.8)	3.9	(2.6-5.4)	.003
Size at visit 2					
HC, cm	17.1	(16.3-18.2)	18.5	(14.8-21.9)	.36
BPD, cm	4.7	(4.5-5)	–	–	–
FL, cm	3.2	(2.9-3.4)	3.4	(2.6-4.2)	.44
AC, cm	13.8	(13-14.8)	16.4	(12.3-19.4)	.13
EFW, g	295	(257-345)	713	(611-831)	<.001
Size at visit 3					
HC, cm	27.9	(27-28.8)	29.4	(27.8-30.8)	.03
BPD, cm	7.6	(7.3-7.9)	–	–	–
FL, cm	5.7	(5.4-5.9)	5.9	(5.6-6.3)	.11
AC, cm	23.9	(22.8-25)	27.4	(25.4-29.4)	<.001
EFW, g	1333	(1203-1495)	1755	(1396-2162)	.01

Abbreviations: AC, abdominal circumference; BPD, biparietal diameter; CRL, crown-rump length; EFW, estimated fetal weight; FL, femur length; HC, head circumference; IQR, interquartile range; MMNP, Mumbai Maternal Nutrition Project. Observed gestational are range were 8 to 14, 15 to 27, and 28 to 36 for visits 1, 2, and 3 respectively. BPD was not reported, as it was measured differently in the two studies.

Fetuses of younger mothers were smaller at the first and second visits (all measures) and had smaller HC, FL, AC, and EFW at visit 3 (Table 2 and Figure 2). Median (IQR) CRL at visit 1 was 2.7 cm (2.2-3.6 cm) in fetuses of mothers ≤22 years, compared with 3.1 (2.4-3.9) cm in fetuses of mothers >27 years. Equivalent data for HC and AC at visit 3 were 27.9 cm (27-28.8 cm) compared with 28 (27.1-28.8) and 23.8 (22.7-25) cm compared with 24.1 (22.9-25.2) cm. Adjusting for possible confounders had little effect on these associations. Maternal age was positively associated with all the longitudinally measured fetal biometry and estimated fetal weight until the third trimester of pregnancy (Table 3). Adjusting for possible confounders did not change these associations.

**Table 3.** Associations Between Maternal Age at Conception (Years) and Fetal Size/Growth During Pregnancy.^a-f^

	Estimate	95% CI	*P* Value
Size at visit 1 (*z*-score)			
CRL	0.01	(0.00-0.02)	**.04**
Size at visit 2 (*z*-score)			
HC	0.04	(0.02-0.06)	**<.001**
BPD	0.02	(0.01-0.04)	**.01**
FL	0.04	(0.02-0.05)	**<.001**
AC	0.03	(0.02-0.05)	**<.001**
EFW	0.04	(0.02-0.06)	**<.001**
Size at visit 3 (*z*-score)			
HC	0.03	(0.01-0.05)	**.002**
BPD	0.02	(−0.01 to 0.04)	.11
FL	0.03	(0.01-0.05)	**.002**
AC	0.04	(0.02-0.06)	**<.001**
EFW	0.04	(0.02-0.06)	**<.001**
Total fetal growth from 0 to 30 weeks per year of maternal age (cm for fetal biometry, g for EFW)			
HC	0.03	(0.02-0.05)	**<.001**
BPD	0.01	(0.00-0.01)	**.005**
FL	0.01	(0.00-0.01)	**.005**
AC	0.04	(0.02-0.06)	**<.001**
EFW	3.51	(2.23-4.79)	**<.001**

Abbreviations: AC, abdominal circumference; BPD, biparietal diameter; CI, confidence interval; CRL, crown-rump length; EFW, estimated fetal weight; FL, femur length; HC, head circumference.

^a^Gestational age- and sex-adjusted *z* scores were used as outcomes for fetal size.

^b^ Data reported are coefficient estimates and 95% confidence intervals.

^c^ *P* Value for linear associations with maternal age as a continuous variable.

^d^Models were adjusted for allocation group, pregnancy intakes of milk and green leafy vegetables, parity, prepregnancy BMI, height, and SLI score.

^e^Models with fetal growth as the outcome were further adjusted for sex, gestational age (GA) and (GA)^2^.

^f^ *P* Values < .05 are in bold types.

Among 969 fetuses with recorded measures of CRL at visit 1, 692 (71%) were of mothers with regular menstrual cycle length. Women with regular menstrual cycle length were older but had similar BMI, SLI score, parity, and educational attainment to women with irregular cycles. Associations between maternal age and CRL were similar to those found in the whole sample (results not shown).

### Pregnancy Outcomes and Newborn Measures

Among the 1360 newborns, 736 (54%) were male. The median gestational age at delivery was 39 weeks (IQR: 38-40 weeks). Of those newborns with known gestational age at birth (n = 1327), 729 (55%) were SGA and 291 were preterm (22%). Maternal age showed an inverted U-shaped relation with gestational age at birth (*P* = .002). Gestational age was lower in women who were ≤19 (median: 39 weeks; IQR: 38-40 weeks), increased in women until the age of 25 (39 weeks; 39-40 weeks), and decreased at higher ages (39 weeks; 37-39 weeks in women >35 years). The odds of preterm delivery increased with maternal age (Table 4). Adjustments for possible confounders did not attenuate the associations. The odds of being SGA decreased with maternal age; however, once SLI score, prepregnancy BMI, height, and parity were included in the model, the association became nonsignificant (Table 4).

**Table 4.** Association Between Maternal Age at Conception and Selected Pregnancy Outcomes.

	Model 1			Model 2		
	Estimate	95% CI	*P* Value	Estimate	95% CI	*P* Value
Preterm^a,b,c,d,e,f^	1.05	1.01-1.09	**.02**	1.06	1.01-1.11	**.01**
SGA^a,b,c,d,e,f^	0.96	0.93-0.99	**.004**	0.99	0.96-1.02	.54
Birth measures (z-scores)						
Weight	0.01	−0.01 to 0.03	.06	-0.01	−0.02 to 0.01	.40
Length	0.01	−0.01 to 0.02	.25	-0.01	−0.02 to 0.01	.28
HC	0.02	0.00-0.03	**.02**	0.00	−0.01 to 0.02	.66
MUAC	0.02	0.00-0.03	**.03**	0.01	−0.01 to 0.02	.44
AC	0.00	−0.01 to 0.01	.98	-0.01	−0.02 to 0.01	.23
CC	0.01	−0.01 to 0.02	.20	-0.01	−0.02 to 0.01	.23
Triceps	0.02	0.00-0.03	**.02**	0.00	−0.01 to 0.02	.56
Subscapular skinfolds	0.02	0.00-0.03	**.03**	0.00	−0.02 to 0.02	.95

Abbreviations: AC, abdominal circumference; BMI, body mass index; CC, chest circumference; CI, confidence interval; HC, head circumference; MUAC, mid-upper arm; SGA, small-for-gestational age; SLI, Standard of Living Index.

^a^ Estimates are odds ratios.

^b^ Model 1 was adjusted for allocation group.

^c^Finally, model 2 was further adjusted for maternal tobacco use, milk and green leafy vegetables intakes in pregnancy, SLI score, parity, height, and prepregnancy BMI.

^d^ Logistic regression models were used when looking at the association between maternal age and preterm birth and small for gestational age.

^e^ Linear regressions were implemented when studying the association between maternal age and newborn measures.

^f^ In bold are the *P* values < .05.

There were positive associations between maternal age and newborn head and mid-upper arm circumferences, and triceps and subscapular skinfolds. There was a positive association, of borderline significance, between maternal age and birth weight. Adjusting for tobacco use, green leafy vegetables, and milk intakes did not influence any of the associations. After adjustments for either parity or prepregnancy BMI, all were nonsignificant. Maternal age was not associated with the other birth measures.

## Discussion

### Main Findings

Among women living in slums in the city of Mumbai, India, and taking part in a randomized controlled nutrition trial, there were marked trends with age in baseline (prepregnancy) maternal body measurements, parity, and socioeconomic status. Younger women were lighter and thinner and had lower parity, educational attainment, and socioeconomic status. Maternal age was related to fetal size throughout pregnancy up to the time of the last scan. Fetuses of younger mothers were smaller in all measurement from the first to the third trimesters. Skinfold measurements and head and mid-upper arm circumferences were smaller in newborns of younger mothers, and the prevalence of SGA babies was higher. Tobacco use, intakes during pregnancy of green leafy vegetables and milk, parity, prepregnancy BMI, height, and weight gain in pregnancy did not influence the associations between maternal age and fetal size/growth, suggesting the possible effect of other factors not captured by these variables. However, the associations between maternal age and newborn measures were attenuated by adjusting for parity, prepregnancy BMI, and height.

### Strengths and Limitations

Strengths of the study were that menstrual period dates were frequently monitored, and additional inclusion criteria were placed on the LMP dates to maximize the accuracy of gestational ages. The estimation of gestational age using the LMP allowed the detection of possible differences in fetal size due to maternal characteristics in early pregnancy. All ultrasound measurements were made by a single-experienced sonologist, reducing “noise” due to interobserver variability. A limitation was that there were relatively small numbers of women in the extreme age groups; the legal age at marriage in India is 18 years, and only married women were recruited in the study, so there were few young adolescents. Information on age at menarche was not collected, and so we were not able to study the effects of gynecological age on fetal and newborn measures. The scheduling of the last scan meant that we could not fully assess associations between maternal age and fetal biometry in the last trimester of pregnancy. The findings in this undernourished population may not be generalizable.

### Interpretation

We were not able to assess growth directly in the first 2 trimesters of pregnancy because of differences in the type of measurements, but smaller CRL at visit 1 (gestational age range 5-13 weeks) in younger mothers suggests slower growth in early gestation. Although many studies have related maternal age to birth outcomes, few have examined maternal age as a predictor of early fetal size. However, 2 previous studies have, like us, shown a positive relationship between maternal age and CRL.^1,5^ The Generation R study in the Netherlands reported a lower degree of tracking of estimated fetal weight (lower correlation coefficients for the association between fetal weight in the second trimester and birth weight) in younger mothers,^21^ but no studies, to our knowledge, have reported associations between maternal age and individual measures of fetal size and growth in different stages of pregnancy. There were significant trends with maternal age for HC, with fetuses and newborns of younger mothers having smaller HC. Because HC is a proxy for brain growth, these associations suggest reduced brain growth in fetuses of younger mothers. The differences in fetal size and growth between younger and older mothers were small, and we do not know whether they are important in terms of later health and function. Lower maternal age has been associated with poorer educational attainment in children, which has been attributed to poorer parenting and nurturing skills among younger mothers.^2^ Our findings suggest that there may be effects of maternal age on fetal neurodevelopment, which bear further investigation.

We do not know the mechanisms for different fetal growth patterns in younger and older women. We speculate that lower fetal growth in younger mothers may reflect less effective nutrient partitioning or transfer of nutrients to the fetus. For example, amino acid kinetics studies suggest that adolescents may be less able than older women to increase their circulating amino acid concentration through synthesis and/or protein breakdown in response to pregnancy.^22,23^ This could reduce the availability of the amino acids to the fetus, especially in late pregnancy when requirements are greatest to support rapid fetal growth. This mechanism does not explain the findings in early pregnancy when fetal nutrient requirements are small. We do not know the consequences, if any, of the maternal age-related differences in fetal growth patterns for fetal development and later health. It has been suggested that fetuses respond differently to undernutrition at different stages of gestation. Depending on the timing, undernutrition can alter fetal growth patterns and specific tissues whose most rapid development coincides with undernutrition.^24^ A study of adult sheep has shown that a limited period of periconceptional undernutrition alters adult body composition and organ weights.^25^ A similar study has shown that undernutrition immediately before or after conception resulted in initial slowing of fetal growth, followed by faster growth in late gestation, and was associated with differences in cardiac weight and hindlimb length at delivery.^26^ Following the children born during the Mumbai trial will represent an opportunity to understand the long-term effects of different fetal growth trajectories in humans.

In contrast to previous studies in high- and low-middle income countries,^2,4^ maternal age was not associated with newborn weight. This could be explained by a lack of power due to relatively small numbers of adolescent pregnancies (≤19 years) or women of advanced age (>35 years).

### Conclusion

Overall, mothers had fetuses that were smaller from 9 to 12 weeks gestation until 28 to 32 weeks. These differences were small and are unlikely to influence clinical practice, but they are consistent and represent new information about the biology of early development. Further studies of the effect of maternal age on fetal growth and of the relationship of different fetal growth trajectories to childhood and later outcomes are needed in other populations.
